# Milieu sélectif de Lowenstein-Jensen à base de vancomycine pour la réduction des contaminations de cultures de mycobactéries par les bactéries sporulantes

**DOI:** 10.11604/pamj.2020.37.345.23016

**Published:** 2020-12-15

**Authors:** Michel Kiréopori Gomgnimbou, Babacar Faye, Juliette Tranchot-Diallo, Antoinette Kaboré, Louis Robert Belem, Dezémon Zingué, Adama Sanou, Hervé Hien, Lassana Sangaré

**Affiliations:** 1 Département des Sciences Biomédicales, Centre Muraz, Bobo-Dioulasso, Burkina Faso,; 2 Laboratoire de Biologie Médicale, Hôpital Militaire de Ouakam, Dakar, Sénégal,; 3 Unité de Formation et de Recherche des Sciences et Techniques, Université Nazi Boni, Bobo-Dioulasso, Burkina-Faso,; 4 Laboratoire Central de Référence, Institut National de Santé Publique, Ouagadougou, Burkina-Faso,; 5 Département de Bactériologie-Virologie, Centre Hospitalier Universitaire Yalgado Ouédraogo, Ouagadougou, Burkina-Faso

**Keywords:** Lowenstein-Jensen, contamination, vancomycine, colistine, nystatine, triméthoprime, complexe *Mycobacterium tuberculosis*, Lowenstein-Jensen, contamination, colistin, nystatin, trimethoprim, vancomycin, Mycobacterium tuberculosis complex

## Abstract

**Introduction:**

le diagnostic de la tuberculose par culture sur milieu solide demeure la méthode de référence malgré le développement de nouvelles méthodes. Cependant, les performances de cette méthode sont entravées par des taux élevés de contamination des cultures dues à des bactéries sporulantes. Ces bactéries sont en général sensibles à la vancomycine et par conséquent à l´association vancomycine, colistine, nystatine et triméthoprime (VCNT). L´objectif de notre étude était donc d´évaluer l´efficacité d´un milieu de culture sélectif de Lowenstein-Jensen (LJ) à base de VCNT pour réduire les contaminations de culture.

**Méthodes:**

des expectorations collectées chez 120 patients tuberculeux et non tuberculeux inclus entre Octobre 2016 et Mai 2017 ont été décontaminées par la méthode modifiée de Petroff. Les culots de décontamination ont été inoculés sur les milieux LJ ordinaires (LJ-MO) et sélectif LJ-VCNT à 10µg/ml de vancomycine. Quinze souches de bactéries sporulantes ont été inoculées sur les mêmes types de milieux afin d´évaluer leur susceptibilité au VNCT.

**Résultats:**

les contaminations des cultures sur le milieu LJ-VCNT à 10µg/ml de vancomycine et sur milieu LJ-MO étaient respectivement de 11,66% (14/120) et de 39,16% (47/120) avec p<0.0001. La susceptibilité des bactéries sporulantes au VCNT diminuait avec l´augmentation de la durée d´incubation des cultures.

**Conclusion:**

le milieu sélectif de L-J VNCT à 10µg/ml de vancomycine a permis une réduction significative du taux de contamination des cultures. Ce milieu pourrait contribuer à l´amélioration de la qualité des cultures des mycobactéries et donc du diagnostic bactériologique de la tuberculose.

## Abstract (en)

**Introduction:**

despite the development of new methods, culture on solid medium is the gold standard for the diagnosis of tuberculosis. However, this method is associated with increased rates of contamination of cultures by spore-forming bacteria. These bacteria are generally sensitive to vancomycin and to a combinsation of vancomycin, colistin, nystatin, and trimethoprim (VCNT). The purpose of this study was to assess the effectiveness of VCNT-based selective Lowenstein-Jensen (LJ) medium in reducing contamination of cultures by spore-forming bacteria.

**Methods:**

sputum samples, collected from the 120 TB and non-TB patients included in the study between October 2016 and May 2017, were decontaminated with the modified Petroff method. Decontamination pellets were inoculated onto conventional LJ media and selective VCNT-based LJ medium containing 10µg/ml vancomycin. Fifteen strains of spore-forming bacteria were inoculated onto the same media in order to assess their sensitivity to VCNT.

**Results:**

the contamination of cultures on VCNT-based LJ medium containing 10µg/ml of vancomycin and LJ medium were 11.66% (14/120) and 39.16% (47/120) with p <0.0001, respectively. Sensitivity of spore-forming bacteria to VCNT decreased with the increasing of culture incubation time.

**Conclusion:**

VCNT-based selective LJ medium containing 10µg/ml vancomycin led to a significant reduction in the rate of culture contamination. This environment could contribute to improve the quality of mycobacterial cultures and thus bacteriological diagnosis of tuberculosis.

## Introduction

La tuberculose, une maladie aussi vieille que l´humanité, constitue toujours un problème de santé publique au niveau mondial. En 2018, le nombre de cas de tuberculose a été estimé à 10 millions avec environ 1,3 million de décès dans le monde [1]. Les objectifs de développement durable pour l´élimination de la tuberculose d´ici 2030 visent à améliorer le diagnostic avec confirmation bactériologique [1]. Parmi les méthodes de diagnostic biologique recommandées par l´OMS, la culture reste une méthode de choix à côté des tests moléculaires rapides (GeneXpert MTB/RIF, Hain MTBDRplus) qui ont permis une réduction considérable des délais de diagnostic (de 4 semaines à 2 heures en moyenne). La culture sur milieu solide Lowenstein-Jensen (LJ), reste d´ailleurs la méthode de référence (le gold standard). En effet, c´est la méthode la plus sensible qui permet l´isolement de souches vivantes de mycobactéries du complexe tuberculosis, d´identifier les espèces, d´évaluer les résistances et de vérifier l´efficacité des traitements [2, 3]. Cependant, l´efficacité de la culture sur milieu solide est beaucoup compromise par des taux élevés de contamination, en moyenne de 31% [4-6] principalement dans les pays à ressources limitées. Ces taux élevés de contamination diminuent la proportion des résultats interprétables, réduisant ainsi les performances diagnostiques de la culture surtout dans les pays à ressources limitées [6].

Les performances de la culture dans les conditions réelles de pratique des pays à ressources limitées, seraient même inférieures à celles de la microscopie. Plusieurs stratégies visant à réduire les contaminations des cultures ont été évaluées. Il s´agit notamment du rinçage de la bouche avec de l´eau ou des antiseptiques avant la collecte des expectorations [7, 8], de l´utilisation de milieux de transport comme OMNIgene Sputum et de l´utilisation de milieux sélectifs supplémentés d´une combinaison d´antibiotiques [9, 10]. Jusqu´à présent, dans la plupart des études, quelle que soit la stratégie évaluée, les taux de contamination ou les taux de cultures faussement négatives demeuraient supérieurs aux taux acceptables. Une récente étude réalisée dans notre laboratoire, qui a caractérisé les contaminants résiduels des cultures a montré qu´il s´agissait dans la majorité des cas de bactéries à gram positif sporulant du genre *Bacillus* [11]. Ces bactéries sont généralement sensibles à la vancomycine [11, 12]. De plus, une évaluation pilote d´un milieu sélectif à base de VCNT une combinaison d´antibiotiques comprenant de la vancomycine, colistine, nystatine, et triméthoprime a montré des résultats encourageants [11]. L´objectif de notre étude était d´évaluer à large échelle l´efficacité du milieu LJ additionné de VCNT sur les contaminants de cultures des mycobactéries. Spécifiquement, il s´agissait de tester l´efficacité du VCNT à réduire les contaminations des cultures, et de déterminer la susceptibilité des bactéries sporulantes aux VCNT.

## Méthodes

**Collecte des échantillons biologiques:** cette étude expérimentale s´est déroulée d´octobre 2016 à mai 2017 à Bobo Dioulasso/Burkina-Faso. Les échantillons (expectorations pulmonaires) de patients suspectés de tuberculose ont été collectés au Centre Régional de Lutte Antituberculeuse (CRLAT) de Bobo-Dioulasso et traités au laboratoire du centre MURAZ. Un échantillon d´expectoration a été collecté par patient inclus. Les critères d´inclusion étaient d´être âgé de 15 ans ou plus, d´avoir été dépisté positif ou négatif à la microscopie au CRLAT et d´avoir consenti à participer à l'étude.

**Préparation des milieux de cultures:** des flacons de VCNT contenant chacun 1,5mg de vancomycine, 3,75mg de colistine, 6250 unités de nystatine et 2,5mg de triméthoprime ont été utilisés pour la supplémentation du milieu LJ ordinaire. Pour la reconstitution de l´antibiotique, 5ml d´eau distillée ont été ajoutée dans chacun des flacons afin de solubiliser l´antibiotique lyophilisé. Cette solution d´antibiotique a été incorporée dans du milieu LJ ordinaire pour l´obtention d´une concentration finale de 10µg/ml de vancomycine (VCNT ref: SR0091E, Oxoid Ltd, wade Road, Basingstoke, Hants, RG24 8PW, UK). Ce choix a été motivé par l´impact négatif observé pour des concentrations à 15 et 30µg/ml de vancomycine sur la viabilité des mycobactéries dans l´étude préliminaire réalisée par Kaboré *et al*. en 2019 [11]. Ces préparations ont été distribuées à raison de 5ml par tube et coagulé pendant 45 minutes à 85°C dans un coagulateur Friocell. Le milieu LJ ordinaire (LJ-MO) a été préparé à partir de poudre de LJ (BIO-RAD, France, réf. 69675) selon les recommandations du fabriquant.

**Culture des mycobactéries:** les échantillons ont été décontaminés par la méthode modifiée de Petroff et consistait brièvement à mélanger un volume égal d´expectoration et de NaOH à 4%. Après une incubation de 20 minutes sous agitateur KHAN, la soude est neutralisée par l´ajout d´eau distillée stérile suivi d´une centrifugation à 3000trs/min pendant 20 minutes. L´étape de neutralisation de la soude est répétée 2 fois et le culot est remis en suspension dans 1ml d´eau distillée stérile. La suspension de culot est utilisée pour l´ensemencement de deux tubes de LJ-MO et deux autres tubes de LJ-VCNT à 10µg/ml de vancomycine en raison de 100µL par tube. Les milieux ensemencés ont été incubés à l´étuve à 37°C, puis examinés au troisième et au septième jour pour identifier essentiellement les cultures contaminées. Ensuite l´observation des milieux a été faite une fois par semaine pendant deux mois afin d´identifier les cultures positives. Les cultures ont été déclarées négatives lorsqu´aucune pousse n´a été observée après deux mois de culture. Une culture était déclarée contaminée dans les deux groupes (LJ-MO et LJ-VCNT) lorsque ses deux tubes étaient contaminés. Les critères d´une culture contaminée étaient: i) tout changement de couleur ou de consistance du milieu ii) toute liquéfaction du milieu de culture iii) et/ou la présence confirmée par coloration au Ziehl Neelsen (ZN) de colonies autres que des mycobactéries.

**Test de susceptibilité des bactéries sporulantes au VCNT:** quinze isolats comprenant trois *B. cereus*, trois *B. licheniformis*, trois *Paenibacillus*, trois *Bacillus* non réactifs, un *B. subtilis*, un *Brevibacillus* ont été utilisés pour la détermination de la susceptibilité des bactéries sporulantes au VCNT. La souche de référence *Escherichia coli* (ATCC25922) et de l´eau distillée stérile ont été utilisées respectivement comme témoin positif et témoin négatif. Les isolats ont été transférés dans un tube en verre contenant environ 3ml d´eau distillée. Ce mélange a été vortexé et la suspension a été calibrée à 0,5 McFarland à l´aide d’un densitomètre cellulaire (580nm) (*DensiCheck, Manifacturing setting and Country*). À partir de la suspension de 0,5 McFarland, l´inoculum a été dilué à 10^-1^et 10^-3^ pour chaque isolat. Un volume de 100µl de chacune de ces dilutions a été inoculé en double sur les milieux LJ sans et avec VCNT à 10µg/mL de vancomycine. Les milieux inoculés ont été examinés aux jours trois, sept, quatorze et vingt et un à la recherche des tubes contaminés. En raison du type de contamination (en nappe) des milieux de L-J, le dénombrement des Unités Formant des Colonies (UFCs) n´était pas praticable. Ainsi, nous avons déterminé les proportions de contaminations en réalisant le ratio des types de bactéries sporulantes qui ont poussées sur les milieux de LJ avec VCNT sur le nombre total de bactéries sporulantes inoculées sur ces milieux.

**Analyse statistique des données:** les données ont été enregistrées dans le logiciel Excel 2013 et l´analyse statistique a été effectuée à l´aide du logiciel Stata SE version 12 software (*College Station, Texas, USA*). Le test de Khi2 a été utilisé pour comparer les taux de contaminations des cultures des expectorations ainsi que les taux de contaminations de culture des bactéries sporulantes sur LJ- VCNT. Le score des Unités Formant des colonies (UFC) des cultures positives sur les deux types milieux ont été comparé. La valeur de p < 0,05 a été considérée comme statistiquement significative.

**Considération éthique:** l´étude a été approuvée par le comité d´éthique institutionnel du Centre MURAZ par la décision n° 2017-03/MS/SG/CM/CEI et le consentement éclairé des patients a été obtenu avant leur inclusion dans l´étude.

## Résultats

**Figure 1:**
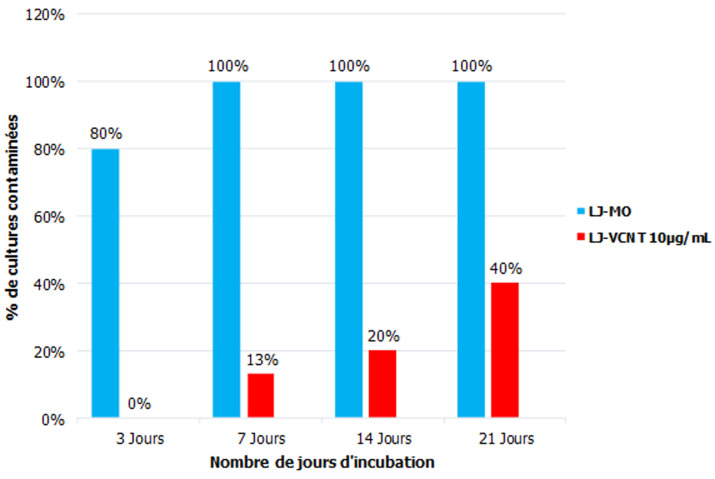
susceptibilité des bactéries sporulantes en fonction du nombre de jours d'incubation

**Figure 2:**
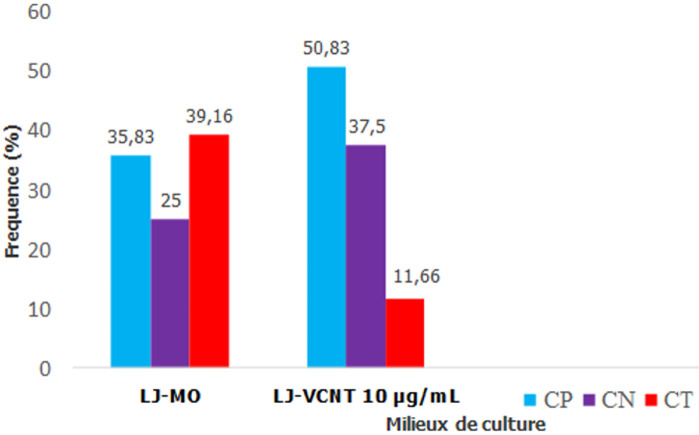
comparaison des cultures d´expectorations sur les milieux LJ-VCNT10µg/ml et LJ-MO

**Susceptibilité des souches contaminant en milieu LJ-VCNT 10µg/ml:** quelle que soit la dilution de l´inoculum, à trois jours d´incubation, 80% (12/15) des *Bacillus* avaient contaminé les milieux de LJ-MO et aucune contamination n´avait été observée sur les milieux de LJ-VCNT à 10µg/ml de vancomycine. Quelle que soit la dilution de l´inoculum, à 7 jours d´incubation, 100% (15/15) des *Bacillus* avaient contaminé tous les milieux de LJ-MO et 13% (2/15) avaient contaminé les milieux de LJ-VCNT à 10µg/ml. A 14 et 21 jours d´incubation, quelle que soit la dilution de l´inoculum, respectivement 20% (3/15) et 40%(6/15) des *Bacillus* avaient contaminé les milieux de LJ-VCNT à 10 µg/ml de vancomycine (Figure 1). Le témoin *E coli* (sensible au VCNT) n´a poussé sur aucun milieu de LJ-VCNT à 10µg/ml de vancomycine (Figure 2).

**Efficacité du LJ-VCNT à réduire les contaminations de culture des expectorations:** nous avons inclus 120 patients suspects de tuberculose parmi lesquels 71 tuberculeux bactériologiquement confirmés (TBC) à la microscopie et 49 microscopies négatives. A l´issu des cultures on a obtenu sur le LJ-MO 35,83% (43/120) de cultures positives; 25% (30/120) de cultures négatives et 39,16% (47/120) de cultures contaminées. Sur le LJ-VCNT 10 µg/ml nous avons obtenu 50,83% (61/120) de cultures positives; 37,5% (45/120) de cultures négatives et 11,66% (14/120) de cultures contaminées. La différence des taux de contamination entre le LJ-VCNT 10µg/ml et le LJ-MO était statistiquement significative (p<0.0001), cf. la Figure 2.

## Discussion

Le milieu LJ-VCNT 10µg/ml de vancomycine a montré une efficacité pour la réduction du taux de contamination des cultures, qui passe de 39% sur le LJ-MO à 11% sur le LJ-VCNT 10µg/ml (p<0.0001). Il a également permis d´améliorer le taux de recouvrement des mycobactéries qui est passé de 36% (LJ-MO) à 51% (LJ-VCNT 10µg/ml). Ceci s´explique par l´action synergique de la vancomycine à 10 µg/ml en association avec les 3 autres antibiotiques, active sur les bactéries à gram positif sporulant, contaminants majeurs des cultures de mycobactéries dans notre laboratoire. Cette efficacité de la vancomycine a été rapportée dans des études similaires, notamment celles de Mathew *et al*. en 2001 en Inde; Chulhun *et al*. en 2002 en Korée; Ramarokoto *et al*. en 2007 à Madagascar qui ont respectivement montré des réductions des taux de contamination de 60% à 20% puis à 4%; de 11 à 5% et de 1.8 à 0,9% [12-14].

Des études ayant portées sur d´autres combinaisons d´antibiotiques, rapportées par Kassaza *et al*. en 2014 en Ouganda; et Okumu *et al*. en 2017 au Kenya ont montré respectivement que l´utilisation de la PANTA (Polymixine B, Amphotericine B, Acide Nalidixic et Trimethoprime et Azlocilline), de selectatab MB, de la Pénicilline G et de la PACT (Polymixine B, Amphotericine Carbenicilline Triméthoprime) ont permis de réduire les contaminations de 32% à 5% , 2% et 9% respectivement et de 15% à 7% pour le PACT [6, 10]. De plus, les taux de contamination sur les milieux sélectifs dans ces études étaient dans les fourchettes acceptables. Leurs résultats pourraient s´expliquer par la différence des types de contaminants retrouvés dans leurs différentes études. En effet, les bactéries identifiées dans l´étude de Kassaza *et al*. par exemple (bactéries à Gram négatif comme *Pseudomonas aeruginosa et Stenotrophomonas maltophilia*) sont naturellement sensibles à la PANTA et à la PACT et pourrait justifier leur résultat. Cependant, au regard de la nature sporulant des contaminants de notre étude, connues pour être résistants à de nombreux agents antimicrobiens y compris la Pénicilline G et les antibiotiques inclus dans la PACT et la PANTA, nous n´aurions pas pu obtenir une meilleure efficacité avec de tels antibiotiques.

La comparaison des scores des cultures positives du LJ-MO et du LJ-VCNT montre qu´il n´y a pas de différence significative (P=0,6), ce qui signifie que le VCNT à 10µg/ml de vancomycine ne compromet pas la viabilité des mycobactéries dans notre étude (données non publiées). Cela corrobore les données trouvées par Kabore *et al*. en 2019 [7]. Notre étude montre que le milieu sélectif de LJ-VCNT 10µg/ml pourrait être utilisé pour améliorer l´isolement des mycobactéries à partir des expectorations surtout dans les laboratoires qui sont confrontés à des taux élevés de contamination liés aux bactéries sporulantes. Ce résultat corrobore et confirme les résultats de l´étude préliminaire réalisé dans notre laboratoire [11]. Toutefois, bien que les taux de contaminations (11%) soient légèrement au-dessus des taux acceptables recommandés par l'Union Internationale contre la Tuberculose et les Maladies Respiratoires (5 à 10%), aucune perspective d´augmentation de la concentration du VCNT n´est envisageable, car il a été montré que l´augmentation de la concentration du VNCT à 15 ou 30µg/ml de vancomycine impacte de manière significative la viabilité des mycobactéries [11]. Toute augmentation aurait donc pour résultat l´accroissement du taux des cultures négatives avec un taux de recouvrement des mycobactéries en baisse.

Pour la susceptibilité des bactéries sporulantes au VCNT, les taux de contamination des milieux de LJ supplémentés de VCNT à 10µg/ml de vancomycine étaient respectivement de 0%, 13%, 20% et 40% après 3, 7, 14 et 21 jours d´incubation. L´augmentation du temps d´incubation semble réduire l´efficacité du milieu de LJ- VCNT à 10µg/ml de vancomycine. Cette réduction de l´efficacité de la vancomycine à long terme (après 14 et 21 jours) pourrait être attribuée non seulement à la reconstitution du produit lyophilisé en solution aqueuse mais aussi à l´incubation des milieux LJ-VCNT à 37°C durant la culture, rendant principalement la vancomycine instable et inactive [15, 16]. Il faut noter qu´en effet, la vancomycine lyophilisée n´est stable qu´entre 20 et 25°C (Pharmacopée américaine, USP, 2018).

Le taux de contamination après 21 jours d´incubation passe à 40%. Bien que ce taux paraisse élevé, il représente néanmoins 60% de réduction de contamination due aux bactéries sporulantes. Etant donné qu´à ce jour, aucune méthode de décontamination ne prend en compte la présence des bactéries sporulantes dans les expectorations, le VCNT pourrait être une alternative en attendant que de meilleures méthodes puissent être développées. Le choix du VCNT revêt un double avantage. Il est largement utilisé en bactériologie médicale et donc son accessibilité serait garantie dans les pays à ressources limitées. Aussi, les expectorations étant polymicrobiens, l´association de la colistine, la nystatine et la triméthoprime dans le VCNT en plus de créer une synergie d´action, permettra de contrôler la contamination d´éventuelles bactéries à Gram négatif, positif ainsi que les champignons.

## Conclusion

Notre étude a confirmé l´efficacité du milieu LJ-VCNT à 10µg/ml de vancomycine à réduire les contaminants majeurs des cultures de mycobactéries sans que la viabilité de ces dernières ne soit compromise. Ce LJ-VCNT pourrait être utilisé comme milieu alternatif pour la culture des mycobactéries afin d´améliorer la qualité des cultures de mycobactéries surtout dans les pays à ressources limitées. Cependant il est nécessaire de poursuivre la mise au point d´un milieu optimal de culture avec de meilleures performances.

### Etat des connaissances sur le sujet

- Les bactéries sporulantes sont les contaminants majeurs des cultures de mycobactéries aux Burkina Faso;
- Pour réduire les contaminations de cultures des mycobactéries, plusieurs stratégies dont celles utilisant des milieux sélectifs à base de combinaison d´antibiotiques ont été évaluées;
- Le milieu LJ supplémenté d´une combinaison d´antibiotique incluant la vancomycine connue pour être efficace contre les bactéries sporulantes n´a jamais été évalué.

### Contribution de notre étude à la connaissance

- Cette étude a montré pour la première fois que les milieux LJ supplémentés de VCNT à 10µg/ml de vancomycine pourraient être utilisés en complément des milieux LJ ordinaires pour la culture des mycobactéries, principalement dans les laboratoires qui sont confrontés à des taux élevés de contamination liés aux bactéries sporulantes;
- Toutefois, l´augmentation du temps d´incubation inhérente à la culture des mycobactéries semble réduire l´efficacité du milieu de LJ supplémentés de VCNT à 10µg/ml de vancomycine.

## Remerciements

Nos remerciements vont au Programme National de Lutte contre la Tuberculose et le Centre Régionale de lutte antituberculeuse pour leur aide dans le processus de collecte des expectorations.
